# The Role of Serum Biomarkers in Predicting Fibrosis Progression in Pediatric and Adult Hepatitis C Virus Chronic Infection

**DOI:** 10.1371/journal.pone.0023218

**Published:** 2011-08-17

**Authors:** Pamela Valva, Paola Casciato, Juan M. Diaz Carrasco, Adrian Gadano, Omar Galdame, María Cristina Galoppo, Eduardo Mullen, Elena De Matteo, María Victoria Preciado

**Affiliations:** 1 Laboratory of Molecular Biology, Pathology Division, Ricardo Gutiérrez Children's Hospital, Buenos Aires, Argentina; 2 Liver Unit, Hospital Italiano de Buenos Aires, Buenos Aires, Argentina; 3 Liver Unit of University of Buenos Aires at Ricardo Gutiérrez Children's Hospital, Buenos Aires, Argentina; 4 Pathology Division, Hospital Italiano de Buenos Aires, Buenos Aires, Argentina; Duke University, United States of America

## Abstract

**Background/Aims:**

Liver biopsy represents the gold standard for damage evaluation, but noninvasive serum markers that mirror liver fibrosis progression are actual goals both in adults and especially in children. The aim was to determine specific serum markers that correlate with liver fibrosis progression during chronic HCV infection.

**Methods:**

Liver biopsies and concomitant serum samples from 22 pediatric and 22 adult HCV patients were analyzed. Histological parameters were evaluated. On serum TGF-ß1, tissue inhibitor of matrix metalloprotein inhibitor-1 (TIMP-1), hyaluronic acid (HA) and aminoterminal peptide of procollagen type III (PIIINP) were tested.

**Results:**

Significant fibrosis (F≥2) and advanced fibrosis (F≥3) represented 64% and 20%, respectively in children; while 54% F≥2 and 23% F≥3 in adults. Hyaluronic acid (*p* = 0.011) and PIIINP (*p* = 0.016) were related to worse fibrosis stages only in adults, along with TIMP-1 (*p* = 0.039) just in children; but TGF-ß1 was associated with mild fibrosis (*p* = 0.022) in adults. The AUROC of TIMP-1 in children to discriminate advanced fibrosis was 0.800 (95%IC 0.598–0.932). In adults, the best AUROCs were that of HA, PIIINP and TGF-ß1 [0.929 (IC95% 0.736–0.994), 0.894 (IC95% 0.689–0.984) and 0.835 (IC95% 0.617–0.957)], respectively. In children, according to the cut off (165.7 ng/mL) value for TIMP-1, biopsies could have been avoided in 72% (18/25). Considering the cut off for HA (109.7 ng/mL), PIIINP (9.1 µg/L), and TGF-ß1 (10,848.3 pg/mL), biopsies could have been avoided in 87% (19/22) of adult patients by using HA and 73% (16/22) using PIIINP or TGF-ß1.

**Conclusions:**

In adults given the diagnostic accuracy of HA, PIIINP, TGF-ß1, their combination may provide a potential useful tool to assess liver fibrosis. This first pediatric study suggests that TIMP-1 is clinically useful for predicting liver fibrosis in HCV patients.

## Introduction

Hepatitis related to HCV is a progressive disease that may result in chronic active hepatitis, cirrhosis, and hepatocellular carcinoma [1], [2], [3]. It represents a global health problem since there is no vaccine currently available and furthermore, liver failure due to chronic hepatitis C (CHC) is one of the most common reasons for liver transplants. Liver disease seems to be milder in children than in adults; however, the natural history of chronic HCV infection acquired in infancy and childhood remains poorly characterized and the long-term outcome of the disease is still a matter of debate [4], [5]. The mechanisms leading to liver cell injury, inflammation, steatosis and fibrosis are still under study. Staging liver fibrosis is considered to be an essential part in the management of patients with CHC, because it provides prognostic information and, in many cases, assists in therapeutic decisions [6]. Although liver biopsy represents the gold standard for evaluating presence, type and stage of liver fibrosis and for characterizing necroinflammation; it remains a costly and invasive procedure with inherent risks. Thus, it cannot be performed frequently to monitor therapeutic outcomes [7], [8]. Moreover, in children, biopsy is still perceived to carry a higher risk of complications, so it is less accepted than in adults. Therefore, developing noninvasive tests that can accurately predict initial disease stage and progression over time represents a high priority and growing medical need [9], [10].

Currently, there are several noninvasive diagnostic methods for determining liver fibrosis that are being validated such as blood markers and imaging methods, but little progress has been achieved in clinical practice [11].

In recent years, many studies have been dedicated to the evaluation of noninvasive indirect serum markers of fibrosis such as serum aminotransferases, aspartate aminotranferase (AST)-to-platelet ratio (APRI) and AST-to-alanine aminotranferase (ALT) ratio (AAR), but they reflect alterations in hepatic function rather than in extracellular matrix metabolism. Since, several HCV reports have described normal aminotransferase levels in about 25%–30% CHC patients, there may be a potential advantage in assessing serum direct fibrosis markers that do not involve transaminases [4], [12], [13], [14]. The extracellular matrix remodelling markers represent attractive candidates because they measure directly the fibrogenic process that leads to clinical complications [15]. These markers include several glycoproteins (hyaluronan, laminin etc), the collagen family (procollagen III, type IV collagen and type IV collagen 7s domain), collagenases and their inhibitors (metalloproteinases and tissue inhibitors of metalloproteinases) and a number of cytokines involved in the fibrogenic process (in particular TGF-ß1). These have been analysed individually as well as combined, in order to assess severity and progression of hepatic fibrosis and to follow up changes related to viral treatment [16], [17], [18], [19], [20], [21], [22], [23].

On the other hand, many authors have combined several biochemical and clinical data (i.e. Fib-4, Forns, Fibrotest) to predict fibrosis stages, but others have brought these together with serum fibrosis markers (i.e. Hepascore, SHASTA, Fibrometer) to do so. [24], [25], [26]. However, this calculation system is tough and complicated to perform routinely in every case.

Finally, considering that most noninvasive approaches for evaluating liver fibrosis have been performed in adults and taking into account the above mentioned considerations about biopsies, there is a clear need to assess these markers in children. Hence, the purpose of our study was to evaluate the presence of a pro-fibrogenic cytokine (TGF-ß1) and matrix deposition markers [hyaluronic acid (HA), type III procollagen amino-terminal peptide (PIIINP) and tissue inhibitor of matrix metalloprotein inhibitor-1 (TIMP-1)] that correlate with liver injury during chronic hepatitis C virus infection, in a cohort of pediatric and adult patients.

## Methods

### Patients and samples

Twenty two pediatric patients with chronic HCV infection (8 male, 14 female; range of age at biopsy: 1–17 years, median: 8 years) who attended the Ricardo Gutierrez Children's Hospital; and 22 adult patients (13 male, 9 female; range of age at biopsy: 38–74 years, median: 51 years) from Hospital Italiano de Buenos Aires were enrolled in the present study.

Diagnosis was based on the presence of anti-HCV antibodies in serum at or after 18 months of age and HCV RNA in plasma at one or more separate occasions. Patients had no other causes of liver disease, autoimmune or metabolic disorders, hepatocellular carcinoma and coinfection with hepatitis B virus and/or human immunodeficiency virus. In adult cases, patients with a history of habitual alcohol consumption were excluded (>80 g/day for men and >60 g/day for women). Patients were naïve of treatment. This study has the approval of the Institutional Review Board and the Ethics Board of both Ricardo Gutierrez Children Hospital and Hospital Italiano de Buenos Aires and is also in accordance with the Helsinki Declaration of 1975, as revised in 1983. A written informed consent was obtained from all the included adult patients and from parents of pediatric patients after the nature of the procedure had been fully explained.

Formalin-fixed paraffin-embedded liver biopsies and serum samples at time of biopsy were used for histological and serological analysis, respectively. Histological sections were evaluated by two independent pathologists in a blind manner. Fibrosis staging were semiquantitatively assessed according to the METAVIR system [27]. Serum samples were stored frozen at −80°C. Serum AST and ALT levels, HCV viral load and genotype, smoking status and alcohol consumption were obtained from clinical records. In pediatric patients, normal ALT and AST levels were ≤32 and ≤48 IU/L, respectively. In adult patients, normal ALT and AST levels were ≤40 and ≤42 IU/L, respectively. In two pediatric cases, more than one sample was analyzed. As controls, serum samples from pediatric (n = 9) and adult (n = 9) healthy subjects without known systemic or liver disease and with normal biological liver test as well as absence of anti-HCV antibodies, were included.

In adult cases, liver samples were not obtained from patients diagnosed as having liver cirrhosis based on clinical, biochemical and imaging findings. Although, there are no pediatric specific guidelines about the need for and timing of a liver biopsy in children, the probability of a child undergoing liver biopsy in this study reflected the current practice at our centre, which is based mostly on the national experts consensus [28].

### Quantitative measurement of human TGF-ß1, TIMP-1, HA and PIIINP

Serum TGF-ß1 and TIMP-1 were determined by commercial quantitative sandwich enzyme immunoassay technique (Quantikine, R&D System Inc) according to the manufacturer's instructions. Serum concentration for each marker was determined from the constructed standard curves. Serum TGF-ß1 was expressed as pg/mL and TIMP-1 as ng/mL.

Serum HA levels were assessed by ELISA (Corgenix) according to the manufacturer's instructions. The HA test kit is an enzyme-linked binding protein assay that uses a capture molecule known as hyaluronic acid binding protein (HABP) and an enzyme-conjugated version of HABP. Hyaluronic acid levels in patient and control samples were determined from the constructed reference curve and expressed as ng/mL.

The levels of PIIINP were measured using a commercial competitive radioimmunoassay technique (UNniQ, Orion Diagnostica) according to manufacture's instructions. Concentrations of PIIINP (µg/L) were obtained from a calibration curve.

Each serum marker concentration was assessed in duplicate.

Operators who perform the laboratory tests were blinded for patient's clinical and histological data.

### Statistical analysis

Statistical analysis was performed using GraphPad InStat software, version 3.05. To compare the means between groups, ANOVA or Student's t test were performed. To determine differences between groups not normally distributed, medians were compared using the Mann-Whitney U test or Kruskal Wallis test. Pearson's correlation coefficient was used to measure the degree of association between continuous, normally distributed variables. The degree of association between non-normally distributed variables was assessed using Spearman's nonparametric correlation. To compare categorical variables Fisher's exact Test was applied. P values<0.05 were considered statistically significant. Results of serum fibrosis markers were expressed as box plots.

To assess the ability of the four serum fibrosis markers for differentiating significant (F≥2) and advanced fibrosis (F≥3), we calculated the sensitivity and the specificity for each value of each fibrosis marker and then constructed receiver operating characteristic (ROC) curves by plotting the sensitivity against the reverse specificity at each value. The diagnostic value of each serum marker was assessed by the area under the ROC (AUROC). AUROC of 1.0 is characteristic of an ideal test, whereas 0.5 indicates a test of no diagnostic value. We determined the cut-off value for the diagnosis, as the maximal value at the sum of the sensitivity (Se) and specificity (Sp). The diagnostic accuracy was calculated by sensitivity, specificity and positive and negative predictive values, considering significant and advanced fibrosis of the disease. Area under the ROC, cut off values, positive predictive values (PPV) and negative predictive values (NPV) were determined using the MedCalc demo statistical software (Mariakerke, Belgium).

Due to the heterogeneous distribution of fibrosis stages on liver biopsies, we applied the DANA method described by Poynard et al [29]. The DANA is an index for standardizing comparisons in order to transform any different fibrosis prevalence profile into a homogeneous distribution of fibrosis stages from F0 to F4, as defined by a prevalence of 0.20 for each of the five METAVIR stages (standard prevalence). The DANA method was applied for each biological non-invasive test. The formula to calculate the adjusted AUROC (AdAUROC) according to a uniform DANA of 2.5 was ObservedAUROC+(0.1056) (2.5-ObservedDANA). Observed AUROC represents the AUROC of the studied group [29].

Finally, we calculated the percentage of patients in whom the results of each serum marker could have avoided the biopsy. Patients correctly classified [true positive (TP)+true negatives (TN)] by a certain test would not have needed the biopsy procedure.

## Results

### 1. Clinical and liver biopsy findings

Clinical, virological, and histological features of patients are described in Table 1 (pediatric patients) and Table 2 (adult patients).

**Table 1:** Clinical, virological and histological features of HCV pediatric patients.

		Clinical and serological characteristics						Histological characteristics			
PediatricPatients		Sex	Ages(ys)	Risk factor for HCV infection	Genotype	Transaminases		Knodell	Lymphoid Follicle	Bile duct damage	Steatosis
						AST (U/L)	ALT (U/L)				
1		M	13	T	1a	47	43	8(5+3)	no	no	absent
2		F	14	T	1b	55	86	11(9+2)	no	no	minimal
3		F	4	V	1a/c	46	34	10(7+3)	yes	yes	absent
4		F	17	T	1a/c	39	43	8(4+4)	no	yes	moderate
5		M	4	V	1a/c	84	97	10(9+1)	yes	yes	severe
6	BxI	F	6	V	1a/c	13	11	7(3+4)	no	no	absent
	BxII		13			23	21	8(5+3)	no	yes	minimal
7		F	16	Unknown	1a/c	30	41	12(10+2)	yes	yes	minimal
8	BxI	M	3	V	1a/c	71	91	6(5+1)	yes	yes	minimal
	BxII		6			314	364	11(8+3)	no	yes	severe
	BxIII		13			225	260	21(16+5)	no	yes	moderate
9		F	6	V	1a/c	35	50	8(7+1)	yes	yes	minimal
10		F	8	Unknown	1a	41	38	9(7+2)	yes	yes	absent
11		M	13	Unknown	ND	56	71	10(7+3)	no	yes	absent
12		F	17	V	1a/c	21	11	6(3+3)	no	yes	minimal
13		F	3	V	1a/c	84	137	6(5+1)	no	yes	moderate
14		F	3	V	1b	65	75	9(6+3)	yes	yes	moderate
15		F	1	V	4	57	33	11(7+4)	yes	yes	absent
16		F	17	T	1a/c	22	16	10(7+3)	no	yes	minimal
17		M	1	T	1b	159	213	14(11+3)	yes	yes	minimal
18		F	8	T	1b	10	12	6(5+1)	no	no	absent
19		M	15	T	ND	58	76	16(10+6)	no	yes	absent
20		F	6	V	1a/c	56	55	6(5+1)	no	yes	severe
21		M	15	T	1b	20	24	13(10+3)	yes	yes	absent
22		M	12	Unknown	1a/c	83	113	11 (8+3)	no	yes	minimal

F: female, M: male. ND: not determined Bx I, Bx II, Bx III denote: multiple liver biopsies. Risk factor for HCV infection: T: transfusion, V: maternal HCV infection.

ALT: alanine aminotransferase; AST: aspartate aminotransferase. Normal ALT and AST levels were ≤32 and ≤48 IU/L, respectively when test was done at 37°C.

**Table 2:** Clinical, virological and histological features of HCV adult patients.

	Clinical and serological characteristics						Histological characteristics*			
AdultPatients	Sex	Ages(ys)	Risk factor for HCV infection	Genotype	Transaminases		Knodell	Lymphoid Follicle	Bile duct damage	Steatosis
					AST (U/L)	ALT (U/L)				
1	M	38	Unknown	1b	82	89	6(5+1)	yes	yes	absent
2	F	52	T	1b	45	52	8(6+2)	yes	yes	minimal
3	M	42	Unknown	1a	44	56	9(8+1)	yes	yes	absent
4	F	62	T	1a	42	32	11(7+4)	no	yes	moderate
5	M	48	DA	1b	40	63	10(7+3)	no	yes	severe
6	M	40	DA	1a	34	45	10(6+4)	yes	yes	moderate
7	M	47	Unknown	1a	29	54	8(6+2)	yes	yes	minimal
8	M	40	DA	2a	63	79	4(4+0)	yes	no	absent
9	M	41	DA	3a	79	86	7(4+3)	no	yes	absent
10	F	61	Unknown	1a	31	28	17(12+5)	yes	yes	absent
11	F	72	Unknown	1a/c	52	71	9(6+3)	yes	yes	minimal
12	F	74	T	1*	106	85	15(12+3)	yes	yes	absent
13	M	62	Unknown	1b	22	32	10(7+3)	no	yes	absent
14	F	55	Unknown	3b	49	60	8(6+2)	yes	yes	severe
15	F	67	Unknown	2a	28	26	6(6+0)	yes	yes	minimal
16	F	41	Unknown	1a	192	105	6(2+4)	yes	yes	absent
17	M	51	OE	1b	65	74	5(4+0)	yes	yes	absent
18	M	51	DA	1a	73	109	10(7+3)	yes	yes	minimal
19	F	67	T	1b	106	103	13(9+4)	yes	yes	absent
20	M	73	Unknown	1b	31	42	8(6+2)	yes	yes	absent
21	M	41	DA	1a	32	50	10(7+3)	yes	yes	severe
22	M	47	DA	3	38	59	3(2+1)	yes	yes	minimal

F: female, M: male.

*Subtype not determined Risk factor for HCV infection: T: transfusion, DA: drug abuse, OE: occupational exposure.

ALT: alanine aminotransferase; AST: aspartate aminotransferase. Normal ALT and AST levels were ≤40 and ≤42 IU/L, respectively when test was done at 37°C.

In both groups genotype 1 was predominant. It was present in 86% of pediatric cases and 77% of adult cases. Only one pediatric patient displayed genotype 4, two adults genotype 2 and three adults genotype 3. The risk factors for HCV infection in pediatric cases were maternal HCV infection in 46% of patients (10/22), transfusion in 36% (8/22) and unknown in 18% (4/22). In adults, seven cases (32%) had a history of injecting drug abuse, one case (5%) described an occupational exposure to infected blood, four (18%) a transfusion as a risk factor and 10 (45%) an unknown source for transmission. The AST and ALT levels at time of biopsy (considering multiple biopsies from the same patient in 2 pediatric cases) were elevated in 52% (13/25) and 76% (19/25) serum samples of pediatric patients, respectively and in 59% (13/22) and 77% (17/22) serum samples of adult patients as well.

Concerning fibrosis, bridging fibrosis was predominant among studied pediatric biopsies (44%). However, one case harbouring thalassemia displayed complete cirrhosis and one case, with an actual progression of liver disease during follow up, incomplete cirrhosis. In adult cases, the fibrosis profile displayed 32% stage 1, 32% stage 2 and 23% stage 3. Finally, three adult patients showed absence of fibrosis. The prevalence of significant fibrosis (F≥2) and advanced fibrosis (F≥3) in the pediatric cohort were 64% and 20%, respectively; meanwhile it was 54% F≥2 and 23% F≥3 in adults.

The comparative statistical analysis of all histological parameters between adult and pediatric patients studied did not show any significant difference. However, it should be taken into account that adult cases with liver cirrhosis based on clinical, biochemical and imaging findings were not biopsied.

Aminotransferase values showed no statistical correlation with fibrosis stages (AST r = 0.04, *p* = 0.85 and ALT r = 0.08, *p* = 0.70 in pediatrics; AST r = 0.14, *p* = 0.50 and ALT r = 0.05, *p* = 0.81 in adults), as well as viral load did not correlate with it either (r = 0.55, *p* = 0.12 in pediatric patient, r = 0.26, *p* = 0.31 in adults). Concerning gender, it has no statistically significant difference related neither to advanced nor significant fibrosis in any of the studied series (*p* = 1.0 for F≥3, *p* = 0.23 for F≥2 in pediatric patients; *p* = 0.11 for F≥3, *p* = 0.41 23 for F≥2 in adults. With regards to the smoking status, none of the pediatric patients is a smoker and in the adult cohort, 12 were no smokers, 7 were smokers and there were no available data in the medical records for the other 3. Smoking status showed no statistically significant difference related either to advanced or significant fibrosis in the studied adult series [Adults F≥2 (*p* = 1), Adults F≥3 (*p* = 0.60)].

### 2. Quantitative measurement of TGF-ß1, TIMP-1, HA and PIIINP

Serum concentrations of studied markers were compared between HCV patients and control healthy subjects. In chronic HCV patients TGF-ß1, TIMP-1, HA and PIIINP levels were higher than in controls (Table 3).

**Table 3:** TGF-ß1, TIMP-1, HA and PIIINP levels in chronic HCV patients vs healthy subjects.

	Pediatrics			Adults		
	Healthy*	HCV+*	*P* value	Healthy*	HCV+*	*P* value
**TGF-ß1** (pg/ml)	2,866	12,178	<0.0001	5,257	12,384	<0.0001
	(770.3–7,125)	(1,400–24,407)		(1,566–7,898)	(2,151–25,679)	
**TIMP-1** (ng/ml)	79.75	142.9	0.0029	114.9	302.6	<0.0001
	(53.98–120.2)	(71.66–845.2)		(92.58–181.1)	(113.5–977.5)	
**HA** (ng/ml)	6.36	10.22	0.03	10.22	80.12	<0.0001
	(0–11.03)	(2.67–228.2)		(4.51–48.41)	(7.90–2,225)	
**PIIINP** (µg/L)	11.43	12.37	0.04	5.54	9.58	0.0004
	(8.64–13.65)	(6.91–27.77)		(3.30–6.61)	(3.41–39.42)	

*Results are expressed as median (min-max).

**Figure 1:**
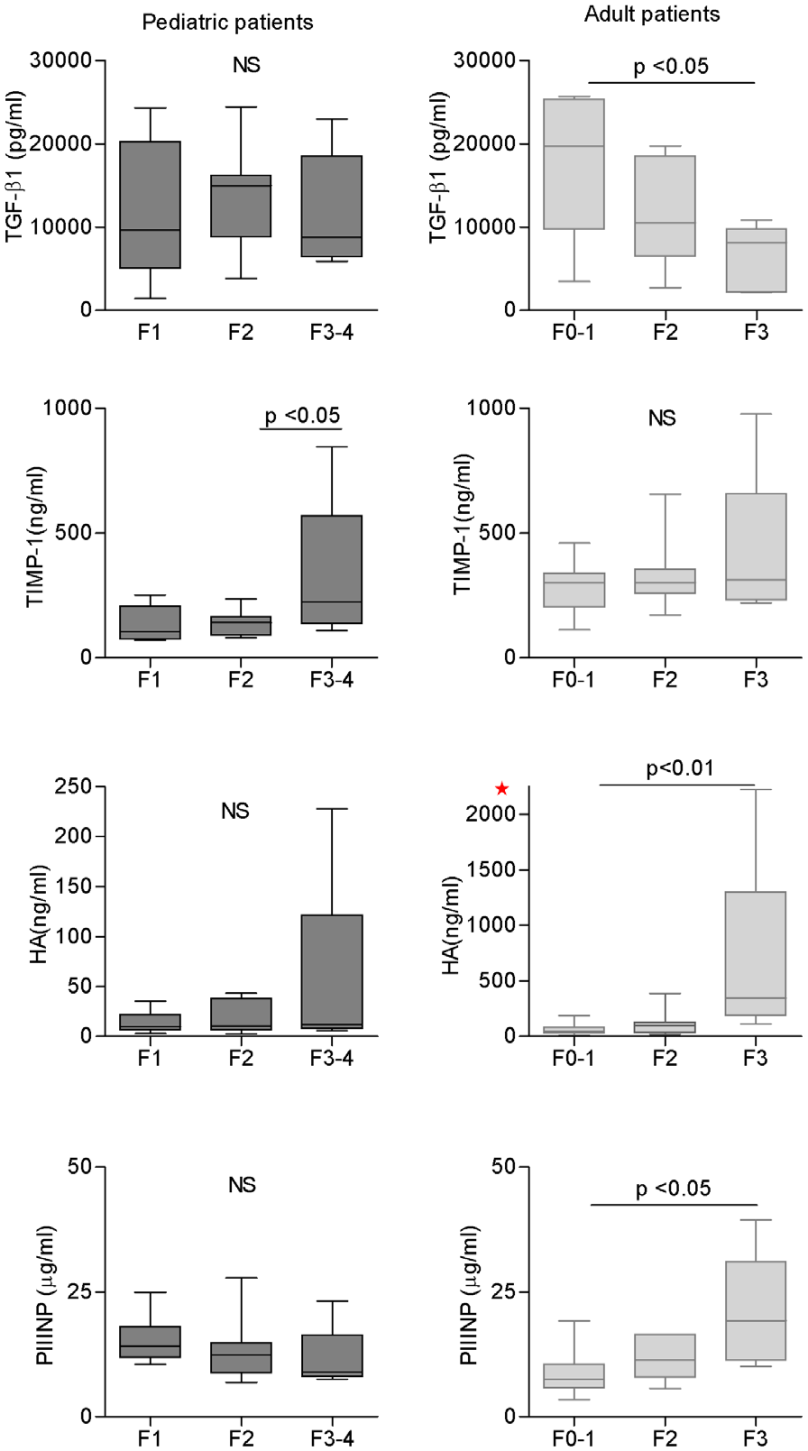
Serum markers related to fibrosis stages in chronic hepatitis C patients. A) TGF-ß1, B) TIMP-1, C) HA, D) PIIINP. Horizontal lines inside each box represent the median, and the lower and upper borders of the box encompass the interquartile range. The vertical lines from the ends of each box encompass the extreme data points. Fibrosis stages according to METAVIR. *NS*: no significant. * Note: different scale compared with pediatric values.

Serum TGF-ß1 showed no statistically significant differences among fibrosis stages in pediatric patients; however it was associated with mild fibrosis (*p* = 0.022) in adults (Figure 1). Higher values of TIMP-1 and HA were observed in both pediatric and adult patients with worse fibrosis stages. The same pattern was observed with PIIINP in adults, but not in children (Figure 1). However, the differences among fibrosis stages were significant for HA (*p* = 0.011) and PIIINP (*p* = 0.016) only in adults, along with TIMP-1 (*p* = 0.039) just in children.

Interestingly, the uppermost values for HA (228.2 ng/mL) and TIMP-1 (845.2 ng/mL) in children correspond to a patient with complete cirrhosis. In adults, the extremely elevated HA value (2,225 ng/mL; result confirmed in three independent assay) and the TIMP-1 (977.5 ng/mL) and PIIINP (39.42 µg/L) uppermost values correspond to the case with stage 4 fibrosis according to modified Knodell scoring system.

### 3. Diagnostic performance of serum markers for significant and advanced fibrosis

**Figure 2:**
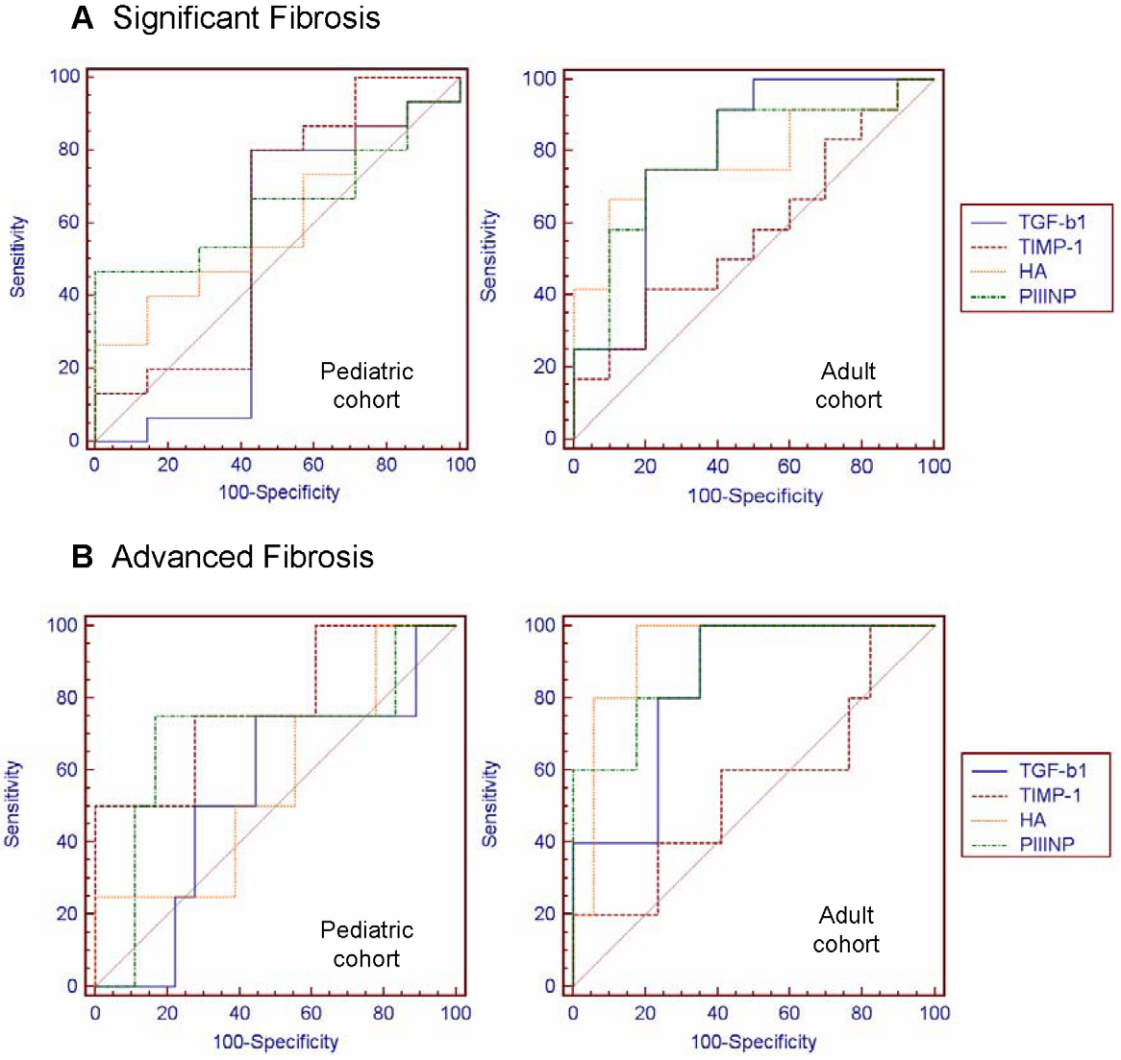
AUROC of TGF-ß1, TIMP-1, HA and PIIINP in pediatric and adult cohorts A) for significant and B) for advanced fibrosis.

The efficiency of these markers to differentiate significant (F≥2) and advanced fibrosis (F≥3) were evaluated using ROC (Figure 2). Table 4 showed the best AUROC for the four serum fibrosis markers. These AUROCs had low but similar diagnostic accuracies for significant fibrosis. In the pediatrics cohort, the best AUROC was that of TIMP-1, which discriminates advanced fibrosis from other stages, with an AUROC of 0.800. On the other hand, in adults, the best AUROCs were that of HA, PIIINP and TGF-ß1 (0.929, 0.894 and 0.835, respectively). For most markers, the accuracy for discriminating significant fibrosis was lower than that for discriminating advanced fibrosis.

**Table 4:** AUROC for significant and advance fibrosis.

	Significant fibrosis (F≥2)			Advanced fibrosis (F≥3)	
	AUROC	95% CI	AdAUROC	AUROC	95% CI
**PEDIATRICS**					
TGF-ß1	0.549	0.339–0.746	0.661	0.570	0.359–0.764
TIMP-1	0.625	0.411–0.809	0.737	0.800	0.593–0.932
HA	0.585	0.368–0.780	0.697	0.562	0.347–0.762
PIIINP	0.648	0.417–0.836	0.760	0.694	0.464–0.870
**ADULTS**					
TGF-ß1	0.792	0.567–0.933	0.875	0.835	0.617–0.957
TIMP-1	0.575	0.349–0.780	0.658	0.553	0.329–0.762
HA	0.783	0.558–0.928	0.866	0.929	0.736–0.994
PIIINP	0.792	0.567–0.933	0.875	0.894	0.689–0.984

Standardization of AUROC by DANA method was carried out for each marker. The analysis showed that AdAUROC were always higher than the corresponding AUROC, but these increments were not significant (Table 4)

Cut off values for each marker in both cohorts for significant and advanced fibrosis stages were shown in Table 5. Many fibrosis experts would consider noninvasive tests for fibrosis with an AUROC value of 0.850–0.900 to be as good as liver biopsies for staging fibrosis [9]; however others consider lower AUROC values to be predictors of fibrosis. In this analysis, those markers with AUROCs higher than 0.800 were only considered. The cut off value for pediatric TIMP-1 in advanced fibrosis was 165.7 ng/mL (80% sensibility, 70% specificity). In adults, the cut-off values of HA, PIIINP and TGF-ß1 were 109.7 ng/mL, 9.1 µg/L and 10,848.3 pg/mL respectively. The sensitivity and specificity for these cut off values were 100% and 82.3% for HA, 100% and 64.7% for PIIINP and 100% and 64.7% for TGF-ß1, respectively.

**Table 5:** Diagnostic accuracy of the serum markers measurement for significant and advance fibrosis.

	Significant fibrosis (F≥2)					Advanced fibrosis (F≥3)				
	Cut off	Se%	Sp%	PPV	NPV	Cut off	Se	Sp	PPV	NPV
**PEDIATRICS**										
TGF-ß1 (pg/ml)	6,950.6	87.5	44.4	73.7	66.7	8,755.7	60	70	33.3	87.5
TIMP-1 (ng/ml)	105.4	81.2	55.6	76.5	62.5	**165.7**	**80**	**70**	**40**	**93.3**
HA (ng/ml)	23.9	40	88.9	85.7	47.1	43.4	25	100	100	87
PIIINP (µg/L)	10.1	46.7	100	100	46.7	9.62	75	83.3	50	93.7
**ADULTS**										
TGF-ß1 (pg/ml)	10,848.3	75	80	81.8	72.7	**10,848.3**	**100**	**64.7**	**45.5**	**100**
TIMP-1 (ng/ml)	336.2	41.7	80	71.4	53.3	654.8	20	100	100	81
HA (ng/ml)	103.1	66.7	90	89	62.2	**109.7**	**100**	**82.3**	**62.5**	**100**
PIIINP (µg/L)	9.1	75	80	81.8	72.7	**9.1**	**100**	**64.7**	**45.5**	**100**

Se: sensitivity Sp: specificity PPV: positive predictive value. NPV: negative predictive value.

In bold are shown the parameters for those markers with best AUROC.

Although PIIINP for F≥2 and HA for F≥3 in pediatric patients had an excellent 100% PPV (Table 5), the diagnostic values were quite low (PIIINP AUROC: 0.648 and HA AUROC: 0.562) (Table 4). The same was observed in adults' TIMP-1 for F≥3 (AUROC: 0.553) (Table 4). The NPV was quite low for all the non-invasive markers of F≥2 in children (always <67%), so that significant fibrosis could not be reliably excluded by any of these markers. The noninvasive methods with best AUROCs (TIMP-1 in the pediatric cohort and HA, PIIINP and TGF-ß1 in adults) showed high NPV (>90%) for advanced fibrosis.

In children, according to the cut off values for advanced fibrosis (F≥3), using TIMP-1 as a serum marker, 14 of 20 (70%) patients with F<3 were correctly categorized, while only 1 of 5 (20%) patients with F≥3 were misclassified (40% PPV and 93.3% NPV). Considering the above, biopsies could have been avoided in 72% (18/25) of pediatric patients. In adults, when the cut-off value for HA was applied, 19 patients were correctly classified (5 patients were TP and 14 patients were TN), and 3 patients were misclassified [False Positive (FP)]. Taking into consideration the cut off for PIIINP, 16 patients were correctly identified (5 patients were TP and 11 patients were TN), and 6 patients were misclassified (6 FP). According to the cut off value for TGF-ß1, 16 patients were correctly identified (5 patients were TP and 11 patients were TN), and 6 patients were misclassified (6 FP). Consequently, biopsies could have been avoided in 87% (19/22) of adult patients by using HA and 73% (16/22) using PIIINP or TGF-ß1.

## Discussion

A major clinical challenge is finding the best means for evaluating liver impairment and managing the increasing number of HCV infected patients. Prognosis and treatment of CHC are partly dependent on the assessment of histological activity, namely cell necrosis and inflammation, and on the degree of liver fibrosis. These parameters have so far been provided by liver biopsy, because conventional laboratory tests are unable to precisely evaluate liver lesions. Biopsy, because of its limitations and risks, is no longer considered mandatory as the 1st-line indicator of liver injury in HCV patients [30], [31], [32], [33]. In addition to the risks related to an invasive procedure, liver biopsy has been associated with sampling error mostly due to suboptimal biopsy size [34], [35], [36]. To avoid these pitfalls, several markers have been proposed as noninvasive alternatives for predicting fibrosis; but few, particularly those which combine clinical and biochemical parameters, have been applied to pediatric patients [37], [38].

The key step in the pathophysiology of liver fibrogenesis is the balance between extracellular matrix (ECM) deposition and removal [39]. It is characterized by the activation and proliferation of hepatic stellate cells (HSCs) which undergo a phenotypic switch when exposed to soluble factors including transforming growth factor beta-1 (TGF-ß1) [40], [41], [42]. Hepatic stellate cells secret excess matrix proteins such as collagens, elastin, glycoproteins, and proteoglycans, then matrix degradation, initiated by a family of enzymes called the matrix metalloproteins (MMPs), occurs. MMP are in turn inhibited by tissue inhibitors of metalloproteins (TIMPs) [17], [43], [44].

Hepatitis C virus Core protein is thought to be involved in the disruption of lipid metabolism leading to the production of the pro-inflammatory cytokine TGF-ß1 [45], [46], [47]. Hyaluronic acid (HA) is synthesized and distributed throughout the extracellular space by HSC, therefore its serum levels reflect the activity state of these cells [48]. Tissue inhibitor of matrix metalloprotein inhibitor-1 (TIMP-1) protects collagen from MMP fibrolysis and also inhibits the apoptosis of HSC [49]. On the other hand, initiating events in HSC activation are occurring on a background of progressive disease changes in the surrounding ECM. Over the time, the subendothelial matrix composition changes from one comprised of type IV collagen, heparan sulfate proteoglycan, and laminin to one rich in fibril-forming collagen, like collagen type III [44].

Concerning our results, there are different points to be emphasized. First, the circulating concentration of TGF-ß1, HA, TIMP-1, PIIINP were higher in CHC patients than in healthy control subjects. Second, many markers seemed to be related to liver fibrosis progression, but with different association patterns in the two cohorts: TIMP-1 levels were associated with fibrosis progression in the pediatric cohort and HA, PIIINP and TGF-ß1 in adults.

Many authors have explored these markers as a potential noninvasive tool to predict fibrosis changes. Most of them evaluated HA in adult HCV patients, and many applied a combined panel of TIMP-1, PIIINP and HA named ELF test [16], [17], [21], [23], [50]. This test which has shown good performance in predicting advanced fibrosis, it is not commercially available worldwide [10]. In our analysis, these above mentioned markers seemed to be also related to liver fibrosis progression, but probably not to be applied altogether as a panel. In accordance with the results published by Patel et al [21], Sanvisens et al [18] and Resino et al [50] our adult studied cohort displayed levels of HA strongly associated with advanced stages of liver fibrosis. Furthermore, our findings also go along with Saitou et al [16] and Leroy et al [17] who described that both HA and PIIINP were related to worse fibrosis stages. However, concerning TIMP-1 conflicting results were reported. Leroy et al [17] found association between TIMP-1 levels and fibrosis stage in adults, while Macias et al [24] did not. Interestingly our results were also discordant, since no association was evidenced in adults but it was presented in children. Although there are few data related to TIMP-1 in children as a single marker of liver injury in different hepatic diseases, our results suggested that TIMP-1 would be suitable as a fibrosis marker in HCV children. This observation is in agreement with data reported by Lebensztejn et al on pediatric HBV patients [51], It is worthwhile to note that the highest TIMP-1 values were those of the cirrhosis case, so it would be interesting to analyze more cases from this condition to assess the actual diagnostic accuracy for cirrhosis.

Diagnostic accuracy is evaluated using the area under the ROC curve [30], [31], [32]. In the studied cohort most markers displayed an AUROC increment between significant and advanced fibrosis. According to Leroy et al [17] in our adult cohort HA and PIIINP were also the markers with the best diagnostic accuracy for advanced fibrosis. Moreover, HA showed moderate accuracy for diagnosis of significant fibrosis (AUROC: 0.783), while it seemed to be a very useful method for detection of advanced fibrosis (AUROC: 0.929). This observation is in agreement with the data published by many reports including adult monoinfected as well as HIV/HCV coinfected patients [16], [17], [18], [50]. Many authors have described the reliability of this marker in series of pediatric HBV infected patient as well as non-alcoholic fatty liver disease [51], [52], [53], [54], [55], [56]. Although in our HCV pediatric cohort HA showed 100% specificity in advanced fibrosis, a remarkable point for a diagnostic tool, it is usefulness seemed to be limited. Finally, a novel finding was the TIMP-1 diagnostic accuracy for advanced fibrosis which was described for the first time in HCV pediatric series.

Poynard et al had proposed that for a disease defined as a combination of stages vs. a combination of other stages, the AUROCs for the diagnosis of it must be expressed in a standardized fashion according to the prevalence of a given stage [57]. They suggested to use a uniform distribution with the same prevalence for each stage (corresponding to a difference in DANA of 2.5 fibrosis METAVIR units between F≥2 and F<2). This finding is clinically significant, because it is easier to discuss the apparently discordant results of a given biomarker observed in the literature [29].Through the literature different AUROCs for significant fibrosis concerning several markers were described. Resino et al [50] reported an AUROC for HA of 0.676, while Levoy et al [17] showed a HA AUROC of 0.75 and a PIIINP AUROC of 0.77 and finally, Saitou et al [16] described an AUROC of 0.805 for HA and of 0.747 for PIIINP. Based on their population distribution published data we could assess DANA values and estimate the AdAUROC for HA and PIIINP. When comparing the HA and PIIINP AdAUROC results of our adult series with the above mentioned estimated AdAUROCs; no significant differences were observed. This confirms that although our series is small, the conclusions drawn concerning the usefulness of HA and PIIINP as fibrosis markers, are in accordance to the data published in larger studies.

The facts that both HA and TGF-ß1 have close relationship with stellate cells and that the activation of stellate cells is crucial for developing hepatic fibrosis led to the hypothesis that serum levels of both HA and TGF-ß1 could be significantly associated with fibrosis [18]. However, the relationship between TGF-ß1, a pro-fibrogenic cytokine, and the hepatic fibrosis is not well established. Nelson et al [58] stated that fibrogenesis is a long process and that the level of fibrosis was the summation of all the effects in the past; therefore, active TGF-ß1 at a certain time point might not correlate with the fibrosis score. In this study adult TGF-ß1 levels displayed an inverse relationship with fibrosis stages, where the lower TGF-ß1 values correspond to the worse stages. Moreover, lower levels of TGF-ß1 were detected when serum samples from 4 adult cirrhotic patients were included (data no shown). In accordance with other authors, lower levels of TGF-ß1 might indicate advanced liver fibrosis suggesting that this marker may reflect fibrogenesis rather than fibrosis [59], [60].

The final aim of a serum marker is to find molecules that mirror liver fibrosis progression as an alternative of the biopsy when it is contraindicated. In our cohort, all adult patients with advanced fibrosis were correctly classified according to each cut off by HA, PIIINP and TGF-ß1 markers. A better sorting of the cases should be achieved if these markers were applied sequentially taking into account their NPV and PPV. Given the diagnostic accuracy of HA it would be chosen as the first line assay, then HA values over cut off could be re-evaluated according either PIIINP or TGF-ß1 cut off. Finally, only those cases with values over each marker cut off should not avoid liver biopsy since it is the gold standard method to assess actual damage of liver.

Finally, there are several reports that analyze APRI and AAR as surrogate indirect serum markers of liver fibrosis. When assessed in our cohorts, these approaches did not improve the diagnostic accuracy performance of the proposed selected markers for each group. In adults APRI showed a low performance which not reach the 0.800 AUROC value proposed to be enough for staging fibrosis, while AAR displayed a limited diagnostic value since it only slightly improved the AUROC of TGF-ß1 to predict only advanced fibrosis. (Table S1 and Figure S1). However, in the pediatric series neither APRI nor AAR reached the 0.800 AUROC value, thus TIMP-1 remained to be the best option.

The present study does have several limitations. First, this was in fact a retrospective study, with a quite limited number of cases, so the small sample size makes it difficult to validate the utility of serum markers. However, the results obtained from our adult patients were similar to the ones reported in other larger adult cohorts. Second, due to medical management protocols from our institutions, pediatric patients without liver fibrosis (F0) and adults with cirrhosis or hepatic decompensation were not available for this study. Third, since we did not take into account biopsy length and fragmentation, the potential for sampling error and understaging of fibrosis remains possible.

This study represents the first analysis performed on pediatric chronic HCV patients and the first comparative study between pediatric and adult cohorts related to a pro-fibrogenic cytokine and matrix deposition markers. Our proposal represents a significant step forward because we suggested that these markers of fibrosis could be easily measured by a single assay without need of special equipment and could be simply interpreted. The solely evaluation of TIMP-1 may be enough to predict fibrosis in children. The availability of a single marker test as TIMP-1, avoids the need of not yet accessible complex tests, such as ELF, that require the evaluation of predictive algorithms. Then, pediatric patient with lower levels of TIMP-1 should avoid liver biopsy. Continued monitoring of markers of liver fibrosis in pediatric patients will help to establish their role in predicting clinical outcomes. Serum TIMP-1 levels below the cut off values could act as a surrogate marker of non-advanced liver fibrosis, thus facilitating the clinical management of patients with HCV disease when liver biopsy is unavailable or contraindicated. It would be useful to study larger pediatric cohorts, perhaps in a multicentre study, to validate and confirm our findings. Perhaps if these parameters are validated in the near future, they would be so easy to assess and interpret as are AST and ALT nowadays. In consequence, this approach would be potentially translatable to the bedside.

## Supporting Information

Figure S1 **AUROC of AAR and APRI in pediatric and adult cohorts A) for significant and B) for advanced fibrosis.**
(TIF)

Table S1 AUROC for significant and advance fibrosis.
(DOC)

## References

[1] Murray K, Finn L, Taylor S, Seidel K, Larson A (2005). Liver histology and alanine aminotransferase levels in children and adults with chronic hepatitis C infection.. J Pediatr Gastroenterol Nutr.

[2] Kage M, Fujisawa T, Shiraki K, Tanaka T, Fujisawa T (1997). Pathology of chronic hepatitis C in children. Child Liver Study Group of Japan.. Hepatology.

[3] Badizadegan K, Jonas M, Ott M, Nelson S, Perez-Atayde A (1998). Histopathology of the liver in children with chronic hepatitis C viral infection.. Hepatology.

[4] Jara P, Resti M, Hierro L, Giacchino R, Barbera C (2003). Chronic hepatitis C virus infection in childhood: clinical patterns and evolution in 224 white children.. Clin Infect Dis.

[5] Chen S, Morgan T (2006). The Natural History of Hepatitis C Virus.. Infection Int J Med Sci.

[6] Dienstag J (2002). The role of liver biopsy in chronic hepatitis.. Hepatology.

[7] Bravo A, Sheth S, Chopra S (2001). Liver biopsy.. N Engl J Med.

[8] Thampanitchawong P, Piratvisuth T (1999). Liver biopsy:complications and risk factors.. World J Gastroenterol.

[9] Afdhal N, Nunes D (2004). Evaluation of liver fibrosis: a concise review.. Am J Gastroenterol.

[10] Martínez SM, Crespo G, Navasa M, Forns X (2011). Noninvasive assessment of liver fibrosis.. Hepatology.

[11] Manning D, Afdhal N (2008). Diagnosis and quantitation of fibrosis.. Gastroenterology.

[12] Gismondi M, Turazza E, Grinstein S, Galoppo M, Preciado M (2004). Hepatitis C virus infection in infants and children from Argentina.. J Clin Microbiol.

[13] Sanai F, Benmousa A, Al-Hussaini H, Ashraf S, Alhafi O (2008). Is serum alanine transaminase level a reliable marker of histological disease in chronic hepatitis C infection?. Liver Int.

[14] Sebastiani G, Vario A, Guido M, Alberti A (2008). Performance of noninvasive markers for liver fibrosis is reduced in chronic hepatitis C with normal transaminases.. J Viral Hepat.

[15] Pinzani M, Rombouts K, Colagrande S (2005). Fibrosis in chronic liver diseases: diagnosis and management.. J Hepatol.

[16] Saitou Y, Shiraki K, Yamanaka Y, Yamaguchi Y, Kawakita T (2005). Noninvasive estimation of liver fibrosis and response to interferon therapy by a serum fibrogenesis marker, YKL-40, in patients with HCV-associated liver disease.. World J Gastroenterol.

[17] Leroy V, Monier F, Bottari S, Trocme C, Sturm N (2004). Circulating matrix metalloproteinases 1, 2, 9 and their inhibitors TIMP-1 and TIMP-2 as serum markers of liver fibrosis in patients with chronic hepatitis C: comparison with PIIINP and hyaluronic acid.. Am J Gastroenterol.

[18] Sanvisens A, Serra I, Tural C, Tor J, Ojanguren I (2009). Hyaluronic acid, transforming growth factor-beta1 and hepatic fibrosis in patients with chronic hepatitis C virus and human immunodeficiency virus co-infection.. J Viral Hepat.

[19] Fontana R, Goodman Z, Dienstag J, Bonkovsky H, Naishadham D (2008). Relationship of serum fibrosis markers with liver fibrosis stage and collagen content in patients with advanced chronic hepatitis C.. Hepatology.

[20] Martinez S, Fernández-Varo G, González P, Sampson E, Bruguera M (2011). Assessment of liver fibrosis before and after antiviral therapy by different serum marker panels in patients with chronic hepatitis C.. Aliment Pharmacol Ther.

[21] Patel K, Lajoie A, Heaton S, Pianko S, Behling CA (2003). Clinical use of hyaluronic acid as a predictor of fibrosis change in hepatitis C.. J Gastroenterol Hepatol.

[22] Fontana RJ, Bonkovsky HL, Naishadham D, Dienstag JL, Sterling RK (2009). Serum fibrosis marker levels decrease after successful antiviral treatment in chronic hepatitis C patients with advanced fibrosis.. Clin Gastroenterol Hepatol.

[23] Parkes J, Guha IN, Roderick P, Harris S, Cross R (2011). Enhanced Liver Fibrosis (ELF) test accurately identifies liver fibrosis in patients with chronic hepatitis C.. J Viral Hepat.

[24] Macías J, Mira J, Gilabert I, Neukam K, Roldán C (2011). Combined use of aspartate aminotransferase, platelet count and matrix metalloproteinase 2 measurements to predict liver fibrosis in HIV/hepatitis C virus-coinfected patients.. HIV Med.

[25] Moreno S, García-Samaniego J, Moreno A, Ortega E, Pineda J (2009). Noninvasive diagnosis of liver fibrosis in patients with HIV infection and HCV/HBV co-infection.. Viral Hepat.

[26] Forns X, Ampurdanès S, Llovet J, Aponte J, Quintó L (2002). Identification of chronic hepatitis C patients without hepatic fibrosis by a simple predictive model.. Hepatology.

[27] Theise N, Bordenheimer H, Ferrel L, Burt AD, Portmann BC, Ferrel LD (2007). Acute and chronic viral hepatitis.. MacSweens Pathology of the liver.

[28] Consenso Argentino de Hepatitis C (2007). Documento final Consenso Argentino Hepatitis C, Asociación Argentina para el Estudio de las Enfermedades del Hígado..

[29] Poynard T, Halfon P, Castera L, Munteanu M, Imbert-Bismut F (2007). Standardization of ROC curve areas for diagnostic evaluation of liver fibrosis markers based on prevalences of fibrosis stages.. Clin Chem.

[30] Gebo KA, Herlong HF, Torbenson MS, Jenckes MW, Chander G (2002). Role of liver biopsy in management of chronic hepatitis C: a systematic review.. Hepatology.

[31] Poynard T, Ratziu V, Benhamou Y, Thabut D, J M (2005). Biomarkers as a first-line estimate of injury in chronic liver diseases: time for a moratorium on liver biopsy?. Gastroenterology.

[32] Sebastiani G, Alberti A (2006). Non invasive fibrosis biomarkers reduce but not substitute the need for liver biopsy.. World J Gastroenterol.

[33] Castera L, Pinzani M (2010). Biopsy and non-invasive methods for the diagnosis of liver fibrosis: does it take two to tango?. Gut.

[34] Poynard T, Halfon P, Castera L, Charlotte F, Le Bail B (2007). Variability of the area under the receiver operating characteristic curves in the diagnostic evaluation of liver fibrosis markers: impact of biopsy length and fragmentation.. Aliment Pharmacol Ther.

[35] Colloredo G, Guido M, Sonzogni A, Leandro G (2003). Impact of liver biopsy size on histological evaluation of chronic viral hepatitis: the smaller the sample, the milder the disease.. J Hepatol.

[36] Regev A, Berho M, Jeffers LJ, Milikowski C, Molina EG (2002). Sampling error and intraobserver variation in liver biopsy in patients with chronic HCV infection.. Am J Gastroenterol.

[37] Hermeziu B, Messous D, Fabre M, Munteanu M, Baussan C (2010). Evaluation of FibroTest-ActiTest in children with chronic hepatitis C virus infection.. Gastroenterol Clin Biol.

[38] El-Shabrawi MH, Mohsen NA, Sherif MM, El-Karaksy HM, Abou-Yosef H (2010). Noninvasive assessment of hepatic fibrosis and necroinflammatory activity in Egyptian children with chronic hepatitis C virus infection using FibroTest and ActiTest.. Eur J Gastroenterol Hepatol.

[39] Friedman S (2003). Liver fibrosis: from bench to bedside.. J Hepatol.

[40] Gressner A (1998). The cell biology of liver fibrogenesis - an imbalance of proliferation, growth arrest and apoptosis of myofibroblasts.. Cell Tissue Res.

[41] Gressner A, Weiskirchen R (2006). Modern pathogenetic concepts of liver fibrosis suggest stellate cells and TGF-beta as major players and therapeutic targets.. J Cell Mol Med.

[42] Powell E, Edwards-Smith C, Hay J, Clouston A, Crawford D (2000). Host genetic factors influence disease progression in chronic hepatitis C.. Hepatology.

[43] Goto T, Mikami K, Miura K, Ohshima S, Yoneyama K (2004). Mechanical stretch induces matrix metalloproteinase 1 production in human hepatic stellate cells.. Pathophysiology.

[44] Friedman S (2008). Mechanisms of hepatic fibrogenesis.. Gastroenterology.

[45] Miyoshi H, Moriya K, Tsutsumi T, Shinzawa S, Fujie H (2011). Pathogenesis of lipid metabolism disorder in hepatitis C: Polyunsaturated fatty acids counteract lipid alterations induced by the core protein.. J Hepatol.

[46] Okuda M, Li K, Beard MR, Showalter LA, Scholle F (2002). Mitochondrial injury, oxidative stress, and antioxidant gene expression are induced by hepatitis C virus core protein.. Gastroenterology.

[47] Taniguchi H, Kato N, Otsuka M, Goto T, Yoshida H (2004). Hepatitis C virus core protein upregulates transforming growth factor-beta 1 transcription.. J Med Virol.

[48] Plebani M, Basso D (2007). Non-invasive assessment of chronic liver and gastric diseases. 2007.. Clin Chim Acta.

[49] Murphy FR, Issa R, Zhou X, Ratnarajah S, Nagase H (2002). Inhibition of apoptosis of activated hepatic stellate cells by tissue inhibitor of metalloproteinase-1 is mediated via effects on matrix metalloproteinase inhibition: implications for reversibility of liver fibrosis.. J Biol Chem.

[50] Resino S, Bellón J, Asensio C, Micheloud D, Miralles P (2010). Can serum hyaluronic acid replace simple non-invasive indexes to predict liver fibrosis in HIV/Hepatitis C coinfected patients?. BMC Infect Dis.

[51] Lebensztejn DM, Sobaniec-Lotowska ME, Kaczmarski M, Voelker M, Schuppan D (2006). Matrix-derived serum markers in monitoring liver fibrosis in children with chronic hepatitis B treated with interferon alpha.. World J Gastroenterol.

[52] Nobili V, Alisi A, Torre G, De Vito R, Pietrobattista A (2010). Hyaluronic acid predicts hepatic fibrosis in children with nonalcoholic fatty liver disease.. Transl Res.

[53] Lebensztejn DM, Kaczmarski M, Sobaniec-Łotowska M, Bauer M, Voelker M (2004). Serum laminin-2 and hyaluronan predict severe liver fibrosis in children with chronic hepatitis B.. Hepatology.

[54] Elmetwally IM, Elmahalaway AM, Abuhashem SH, ahmed AM (2009). Determination of serum fibrosis index in patients with chronic hepatitis and its relationship to histological activity index.. Saudi Med J.

[55] Saltik-Temizel IN, Koçak N, Ozen H, Demir H (2004). Serum hyaluronic acid concentrations in children with cirrhosis.. Indian J Gastroenterol.

[56] Hartley JL, Brown RM, Tybulewicz A, Hayes P, Wilson DC (2006). Hyaluronic acid predicts hepatic fibrosis in children with hepatic disease.. J Pediatr Gastroenterol Nutr.

[57] Poynard T, Halfon P, Castera L, Charlotte F, Le Bail B (2007). Variability of the area under the receiver operating characteristic curves in the diagnostic evaluation of liver fibrosis markers: impact of biopsy length and fragmentation.. Aliment Pharmacol Ther.

[58] Nelson DR, Gonzalez-Peralta RP, Qian K, Xu Y, Marousis CG (1997). Transforming growth factor-beta 1 in chronic hepatitis C.. J Viral Hepat.

[59] Soliman GM, Mohammed KA, Taha A, barrack AA (2010). The role of plasma transforming growth factor beta-1 in the development of fibrosis in patient with HCV related steatohepatitis... J Egypt Soc Parasitol.

[60] Rallón NI, Barreiro P, Soriano V, García-Samaniego J, López M, Benito JM (2011). Elevated TGF-β1 levels might protect HCV/ HIV-coinfected patients from liver fibrosis.. Eur J Clin Invest.

