# Supplementary figures and images for: The Role of Serum Biomarkers in Predicting Fibrosis Progression in Pediatric and Adult Hepatitis C Virus Chronic Infection
Source: PLoS One. 2011 Aug 17;6(8):e23218. doi: 10.1371/journal.pone.0023218 (PMC3157356; doi:10.1371/journal.pone.0023218)

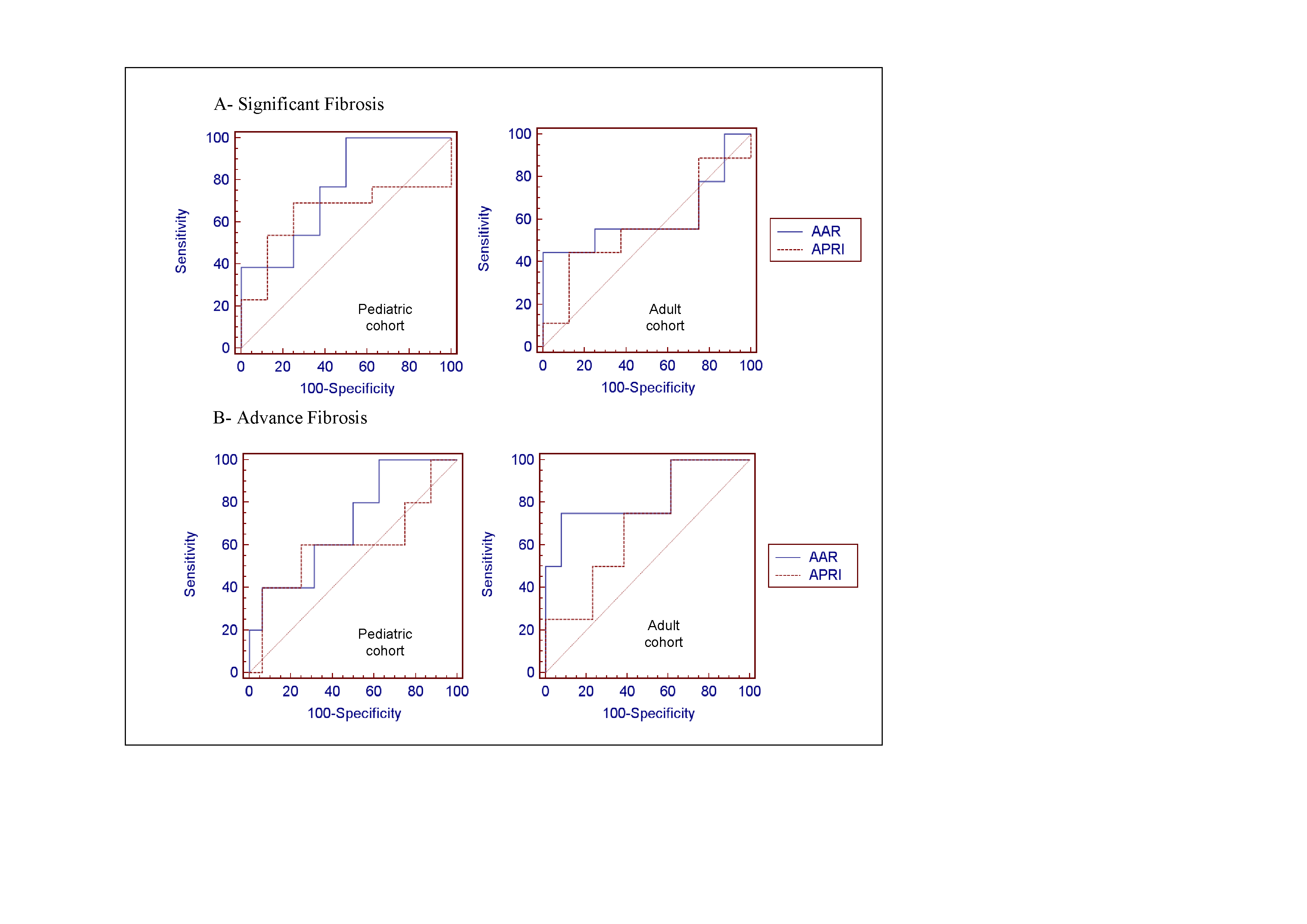

Supplement: Figure S1 — AUROC of AAR and APRI in pediatric and adult cohorts A) for significant and B) for advanced fibrosis. (TIF) [file pone.0023218.s001.tif]
