# Supplementary material for: The Role of Serum Biomarkers in Predicting Fibrosis Progression in Pediatric and Adult Hepatitis C Virus Chronic Infection
Source: PLoS One. 2011 Aug 17;6(8):e23218. doi: 10.1371/journal.pone.0023218 (PMC3157356; doi:10.1371/journal.pone.0023218)
Supplement: Table S1 — AUROC for significant and advance fibrosis. (DOC) [file pone.0023218.s002.doc]

Supplemental material Table I: AUROC for significant and advance fibrosis.

|  | **Significant fibrosis (F≥2)** | | | **Advanced fibrosis (F≥3)** | |
| --- | --- | --- | --- | --- | --- |
|  | **AUROC** | **95% CI** | **AdAUROC** | **AUROC** | **95% CI** |
| **PEDIATRICS** |  |  |  |  |  |
| AAR | 0.774 | 0.564-0.916 | 0.886 | 0.650 | 0.435-0.828 |
| APRI | 0.644 | 0.408-0.838 | 0.756 | 0.600 | 0.366-0.804 |
| **ADULTS** |  |  |  |  |  |
| AAR | 0.567 | 0.341-0.773 | 0.636 | 0.859 | 0.645-0.969 |
| APRI | 0.556 | 0.300-0.791 | 0.625 | 0.692 | 0.427-0.888 |
